# Adolescents’ Perspectives on Facilitators and Barriers to Social Health in the Family: A Qualitative Study

**DOI:** 10.34172/aim.2023.60

**Published:** 2023-07-01

**Authors:** Mahnaz Solhi, Ali Taghipour, Mehrsadat Mahdizadeh

**Affiliations:** ^1^Air Pollution Research Center, Iran University of Medical Sciences, Tehran, Iran; ^2^Department of Epidemiology, School of Health, Mashhad University of Medical Sciences, Mashhad, Iran; ^3^Department of Health Education and Health Promotion, School of Health, Mashhad University of Medical Sciences, Mashhad, Iran

**Keywords:** Adolescent, Adolescent health, Family, Healthy family, Qualitative study, Social health

## Abstract

**Background:**

Family plays the most fundamental role in the adolescent’s health. A deep understanding of family characteristics, beliefs, and function about the adolescent social health provides a framework, relying on which one can perceive how this dimension of health is developed and promoted in this setting. In this study, we aimed to understand the family context that facilitates or limits adolescent social health.

**Methods:**

Fifty-four adolescents and fifteen parents participated using a purposive sampling method. The findings were collected through semi-structured interviews and group discussions. The data was analyzed through conventional content analysis by the MAXQDA10 software.

**Results:**

Healthy and unhealthy family reactions are the two main categories that facilitate and limit the adolescents’ social health. Sub-categories of healthy reactions included effective guidance, cultural safeguard, and accountable interactions. The unhealthy family reactions included sub-categories of poor intergenerational perception and passive parenting.

**Conclusion:**

Our findings revealed that the family context of the adolescent’s social health ranged from healthy to unhealthy responses. These results can contribute to improving and designing interventions for promoting the adolescent’s social health. It is essential for policymakers and health experts to pay attention to the family empowerment approaches.

## Introduction

 Family factors are known as one of the determinants of health in many countries and cultures.^1^ Adolescents’ development and health are affected by various settings, in which their growth and development occur, including the family, school, and society. All these social settings have a reciprocal impact on adolescent health,^2^ but the family, as the first place of socialization, plays a major role in this regard.^2,3^

 Family plays a key role in the formation of concepts of health and disease, and the normal and abnormal behaviors of its members.^4^ The type of parental interaction with children in the family has special behavioral consequences on their health. Family with its performance can provide a context for the growth of differentiated individuals with healthy function, completely dependent individuals with extensive problems, or individuals falling between these two extremes. Via its functioning, family can also be a factor in strengthening or collapsing the relationships between its members.^5,6^ Inappropriate parental behaviors in the family lead to the formation of inappropriate and antisocial behaviors in adolescents.^7^ On the other hand, normal family functioning and a healthy family environment can ensure the growth of the adolescents’ personality and health.^8^

 Evidence indicates that, nowadays, families have many problems, including parental educational behavior that is affected by various factors. Rapid progress resulting from industrialization has led to extensive variations in family characteristics.^9,10^ These extensive variations in society and its cultural context have led to emergence of interactions, demands between family members, especially adolescents and parents. In such situations, it is difficult to raise children.^11^ Evidence reveals that parents and adolescents in the family are dealing with problems that affect adolescent health.^12^ Through its social, cultural, and material resources, family could play a facilitating or inhibitive role in maintaining and promoting the health of its members with the highest impact.^13^

 Any attempt to achieve health, especially the social health of adolescents and their interactions in the family context, requires a deep understanding and finding solutions for its promotion. Perceiving the experiences of parent-adolescent interactions in the family can help identify the major challenges and strategies in order to provide a proper family context for promoting the social health of adolescents. This research is a qualitative study to achieve an in-depth understanding of the facilitators and barriers of adolescents’ social health in the family context for planning and implementation of family health interventions.

## Materials and Methods

###  Research Design

 In this qualitative study, inductive content analysis was used to recognize the experiences and viewpoints of the study participants. This study used the conventional content analysis method, which helps to extract categories directly from the data.^14,15^

###  Study Participants

 Our participants were 54 adolescents (28 girls and 26 boys) aged 13‒18 years and 15 parents (7 mothers and 8 fathers) aged 28‒54 years. The participants were selected from different areas, including schools, parks, cultural centers, and other places in 2016‒2017 in Mashhad, Iran. Mashhad is the capital of the Khorasan Razavi province in eastern Iran which is one of the major provinces in Iran with considerable racial, cultural, social, and economic diversity.

###  Procedure

 The study participants were chosen by purposive sampling with maximum variations based on demographic characteristics and location differences. In qualitative researches, sampling is undertaken with maximum variation by purposive sampling. The adequacy of the sample size depended on reaching the data saturation. Data saturation was achieved when the sampling did not provide new data.^16^ In this study, after obtaining data saturation, three interviews were conducted with two female adolescents and one male adolescent to ensure the completion of the data.

 The inclusion criteria included the consent of adolescents and their parents to participate in the study, Iranian nationality, age of 13‒18 years, living with at least one parent, and ability to transfer experiences.

 Data were collected through 41 semi-structured face-to-face interviews with 15 parents, 23 adolescents, and 3 key informants. In addition, four group discussions were conducted with 31 adolescents (17 girls and 14 boys). Field notes were also written to collect the data. Before the interviews and group discussions, the interviewer established face-to-face communication with the participants to coordinate time and place. The interviews and group discussions were conducted in a quiet and appropriate place in schools, public health centers, and cultural centers across the city. Comprehensive information was given to the participants about the study objectives and study methods. The interviews lasted between 50 and 70 minutes and the group discussions lasted between 90 and 140 minutes.

 An interview guideline was developed for this study. Interview guide questions were examined in a pilot study with the participation of four adolescents. Some of the questions were as follows: 1. What are the conditions in your family which you consider inappropriate for your social health? 2. What are the conditions in your family which you consider appropriate for your social health? 3. (Directed towards parents) What do you think is important for adolescent social health in your family? The questions were continued by probing questions such as “why” and “please explain more “ to provide a deeper understanding of the social health in the family context.

###  Data Analysis 

**Figure 1:**
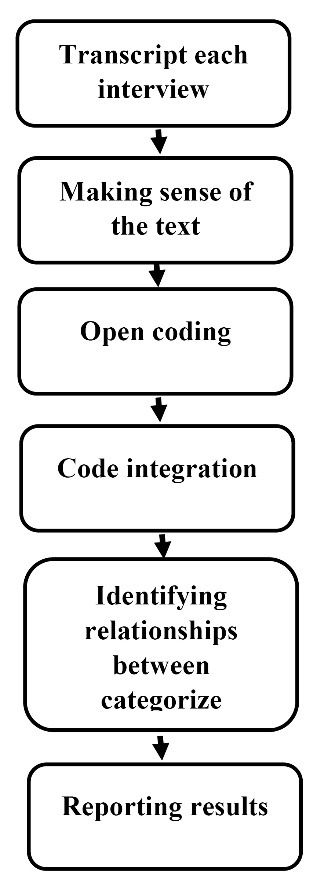
Extraction and Categorization of Codes in the Content Analysis Process

 Data were analyzed using the approach proposed by Graneheim and Lundman.^17^ After recording the interviews and group discussions, each one was listened to several times and then, transcribed by one of the researchers. The MAXQDA 10 software was used for organizing and encoding data. Each interview and group discussion was broken down into semantic units and encoded separately word by word and line by line. The encoding process was done by comparing the codes based on their differences and similarities. The codes were then integrated and classified with respect to their hidden concept. Each category was compared to others and integrated into a more abstract level; the relationship between categories was identified and themes were extracted (Figure 1).

 In this study, the researchers tried to identify their interests in the research topic and avoid prejudice against the participants’ opinions to obtain accurate information based on the participants’ experience. Researchers acquired the necessary skills to conduct a qualitative study by taking a qualitative research method course during their studies and participating in advanced workshops on qualitative research methods.

###  Establishing Rigor of the Study 

 To ensure the scientific precision and validity of data, we used the criteria for evaluating the quality of qualitative studies, based on the guidelines of Lincoln and Guba,^18,19^ which included long-term engagement of the researchers for data collection and analysis. Other criteria that helped to achieve data trustworthiness included detailed reporting of data analyses, peer checking by examining the analysis process by all researchers, and member checking by review of the findings and feedback from participants. Two experts in health and psychology reviewed and confirmed the findings. Furthermore, data were collected in different situations, from different people and using different methods.

## Results

 In this study, 69 individuals participated (See Table 1). Two out of 54 adolescents were living with their father and three adolescents were living with their mother. The other adolescents were living with both parents. The number of family members was between one and six. Parents had elementary to doctoral level of education. Three of the fathers worked in the private sector and others had various governmental jobs, including teachers, doctors, engineers, etc. Also, three mothers were housekeepers and four mothers were employed.

**Table 1:** Characteristic of Participants

**Key Characteristics**	**Gender**	**N**	**Age **
Adolescents	Female	28	Mean = 16.14
	Male	26	Mean = 15.21
Parents	Female	7	Mean = 46.8
	Male	8	Mean = 49.2

 Based on the participants’ experience, the family’s response to adolescent social health ranged from healthy to unhealthy reactions. The sub-categories of healthy reactions included favorable guidance, accountable interactions, and cultural safeguard. The unhealthy family responses had the following sub-categories: poor intergenerational perception and passive parenting. Each of these categories had several sub-categories, which are listed in Table 2.

**Table 2:** Summary of Categories and Sub-categories Extracted from Study Results

**Facilitators**		**Barriers**	
**Category**	**Sub-categories**	**Category**	**Sub-categories**
Healthy reactions	Favorable guidance	Unhealthy reactions	Passive parenting
	Accountable interactions		Poor intergenerational perception
	Cultural safeguard		

###  Healthy Reactions

 Adolescents perceived some family conditions as a healthy family environment. They declared that favorable guidance, accountable relationships, and cultural safeguard facilitate their social health.

###  Favorable Guidance

 Participants expressed the opinion that family guidance is important for their social health. According to the adolescents, the guidance of the family in dealing with problems and risks could help them act effectively in the society and develop their social functioning as a member of the society. In their opinion, these points could provide the basis for their social health. The study participants noted the family’s role in guidance as follows:

 “*… how to defend our rights and control ourselves in any situation. All of this is important and my family guided me about that.”* (Code 1, 18-year-old girl).

 One of the parents who is the father of a 13-year-old adolescent said:

 “*I always talk to my son and I show him the way of life in his own language; you know, when you’re dealing with children, you should talk to them in their own language.”* (Code 49, 56 years old, father of 13-year-old boy)

###  Accountable Interactions

 Family accountable relationships were extracted, including the sub-categories “intimate and reliable relationships”, “family support”, and “family cohesion”. Participants who had experienced warm, intimate, and supportive relationships with family members mentioned that these conditions were positive for their social health.

 “*... I am intimate with my mother; I tell her everything that happens to me. It largely reduces my problems with my friends and those I’m communicating with in the society who have caused me problems...”* (Code 2, 13-year-old girl).

 Intimate relationships between family members created an atmosphere full of trust between adolescents and their parents that helped them have successful experiences in their society in a way that their social health is guaranteed.

 “*I’m very close to my parents. We fully trust each other. This has given me more opportunities to participate in outdoor activities...”* (Code 23, 18-year-old boy)

 The mother of an adolescent boy also said:

 “*It’s necessary to support them. There is a lot of tension in our society. I believe that the home should be good enough to encourage them to come home. We have tried to provide these conditions…”* (Code 6, 39-years-old mother of a 14-year-old boy).

 Some adolescents believed that family cohesion, which could also lead to their support, played a major role in their social health. In this case, they pointed out:

 “*We are always together as a family. My sisters who are now married are always in our home. We are very close. We support each other...”* (Code 11, 15-year-old girl)

###  Cultural Safeguard

 This concept reflects the importance of cultural factors in the family context, including sub-categories of “clarification of norms and values” and “moral and religious orientation.”

 One of the participants said:

 “*My mother does her best to make me a cultured and decent person, so I could be useful for the society. I love the culture of my country and this was the influence of my parents…”* (Code 25, 16-year-old girl)

 According to the experience of the participants, the family plays a major role in clarification and proper transfer of cultural elements, including social values, and following these values would bring them social health. In this regard, the participants said:

 “*It’s important to have a family, through which you can get to know the values better and respect these values...”* (Code 1, 18-year-old girl).

 For the participants, paying attention to morals and religious beliefs in the family was important for the adolescents’ social health. Affection, empathy, and attention to meeting the needs of others were moral qualities. Based on the experience of the participants, commitment to such moral and humanitarian values in the family context can lead to the development of desirable social relationships in the society and bring social health to adolescents.

 “*When I see someone sitting in a corner, I ask him about his problems. I would like to help him in any way. I have learned to behave like this from my parents.”* (Code 21, 16-year-old boy).

 “*In our family, everyone should respect each other. Well, it is very important, it has been mentioned in the Holy Quran as well …”* (Code 5, 14-year-old girl).

###  Unhealthy Reactions

 Unhealthy family responses consisted of two sub-categories of “disturbed intergenerational perception” and “passive parenting”. These conditions hindered the adolescents’ social health.

####  Poor Intergenerational Perception

 The viewpoints and experiences of most of the participants revealed that because of rapid changes in modern life, the families were experiencing conditions which led to incomplete parental understanding of the needs and expectations of adolescents as well as family conflicts. Adolescents experienced these conditions as a barrier to their social health. The participants pointed out these conditions as follows:

 “*My situation now is very different from that of my older siblings. There were no internet and satellite TV channels back then. My parents think that they should behave with me the way they had been treated.”* (Code 42, 17-year-old girl).

 “*Neither do I understand them (my parents), nor do they understand me. Well, their behavior makes me choose my own path in life...”* (Code 34, 16-year-old boy).

####  Passive Parenting

 Passive parenting includes the actions that parents take according to the conditions of the country’s transition, modernity, and the development of communication technologies in their parenting style. Adolescents experience this parenting pattern as a threat to their social health.

 “*Social conditions are entirely different now from when we were adolescents. Things weren’t the same. We try to adapt ourselves to his wishes”* (Code 48, mother of 18-year-old boy).

 One of the parents, on monitoring her child’s function on social media, said:

 “*We can’t limit his use of the internet. Well, for example, he says I’m doing a research for school. What can we do?”* (Code 51, 38 years old, mother of a 13-year-old boy).

 Based on the experience of participants, indifference to adolescent behavior was one of the measures that had a negative impact on their social health. The descriptions are given below: *“… they (my parents) don’t care about what I’m doing. I think they’re indifferent to me. I’m indifferent to them as well.”* (Code 16, 16-year-old boy).

 Parents’ extreme orientation towards religions issues was another action of parents and, based on the participants’ experience; it was an obstacle to their social health. Participants noted:

 “*The same strict religious practices of my parents made me do a lot of things that they don’t like, not listen to their words, and act against their wishes…”* (Code 28, 15-year-old girl).

 A family counselor said:

 “*Some parents are extremely religious in contrast to their children. This has caused a lot of problems …”* (Code 41, 44-year-old man).

 Limiting the adolescents was another strategy that parents used for responding to the need of adolescents for social participation and contact with friends outside home. This action of the parents led to unsafe experiences and family conflicts. Under such circumstances, the adolescents described the family atmosphere as unhealthy and a threat to their social health:

 “*… Sometimes, my parents force me; well that is a big obstacle for us. They’d better let us go out of the house …”* (Code 29, 16-year-old boy).

## Discussion

 This study aimed to understand the family context that facilitates or limits the adolescents’ social health. The results reveal the experiences of participants on the facilitators and barriers to the adolescents’ social health in the family context. The family’s response to adolescent social health ranged from healthy to unhealthy. The results of this study showed that the transition of the country towards urbanization and rapid changes in technology have placed the family in a condition that makes parents use different strategies to meet the social health needs of adolescents. In a healthy family, parents try to optimally respond to the social health needs of their adolescents through accountable interactions, favorable guidance, and cultural safeguard. On the other hand, components of an unhealthy family indicate that parents use parenting strategies such as poor intergenerational perception and passive parenting. These conditions are consequences of industrialization, and do not fulfill the social health needs of adolescents.

 In the present study, accountable interactions were a component of a healthy family that helped adolescent social health. Our findings demonstrated that social relationships were a protective factor for adolescent health, and family relationships were one of the most important factors that could prevent poor health consequences in adolescents.^2^ The children of parents who had a positive parenting style and were aware of their children’s actions were less likely to engage in problematic behaviors and more likely to have better health.^20^ The supportive relationships in the family could lead to adolescents’ resilience to risk factors and reduce the likelihood of adolescent vulnerability to negative experiences. On the other hand, such relationships can promote social, psychological, and cognitive development of adolescents.^4^

 Studies have shown that family conditions, supportive emotional relationships, and family support are adolescents’ health needs.^21,22^ A meta-analysis reported that children living in families with an intimate parenting style as well as balanced control and monitoring and trust in children had the right for freedom and independence and dealt with fewer problems than the families without these conditions.^23^

 According to socialization theories, positive parenting is associated with children’s pro-social behaviors.^24^ Evidence suggests that children who have good mutual relationships with their parents and use balanced and positive parenting styles in the family would have more pro-social relationships.^25^ Spinrad et al highlighted the key role of adults in supporting the development and sustainability of adolescent health.^26^

 Findings have shown that intimate and supportive parents express more positive emotions in interacting with their children and, thus, provide opportunities for effective learning to promote self-regulation of feelings and behaviors for children.^27^ The intimate relationship between parents and children in the family and similar values between adolescents and their parents were associated with further internalization of family and social values, such as the children’s help and respect for others.^28,29^

 Generation gap and its consequences, such as family conflicts and poor understanding of adolescents’ needs, were identified in the experience of participants in the present study. Most adolescents in the present study noted that the parenting style of their parents in these conditions was threatening to their social health. Previous studies have shown that perceived contradictions are associated with a wide range of adverse consequences for children.^10^

 Intergenerational contradictions between parents and children are associated with variables of low culture, low family cohesion, increased parental control in the family, and problematic behaviors of adolescent (e.g. greater externalizing, internalizing and substance use).^30-32^ Studies also revealed that the main motivator of conflict with parents was due to problems such as the time spent outside the house, type of clothing, religious beliefs, and issues related to the adolescence period including independence and parental resistance to their wishes.^33^

 Moreover, a healthy family atmosphere is associated with a high level of pro-social behaviors of adolescents through the parent-child relationship, such as supportiveness and warmth.^34^ Similar to the findings of this study, the results of a previous study indicated that a family with components of affection, encouragement, engagement, teaching, responsiveness, and positive discipline is one of the needs for child well‐being.^35^

###  Strengths and Limitations

 This study on the facilitators and barriers of adolescents’ social health in the family context provides an in-depth understanding for health professionals and other professionals to plan and implement family-based interventions. On the other hand, this study was conducted in one of the big cities of Iran with racial and social diversity, which could increase the generalizability of findings for similar societies. In addition, the results of interviews could be used as basic data for other studies. However, this study had some limitations, such as not interviewing the participants at home, which could provide a better understanding of the family context in developing and promoting the adolescent’s social health. Another limitation of the research was the inability to make continuous contact with the participants due to the reluctance of some participants to provide their phone number.

###  Practical Implications 

 Adolescence is associated with many challenges and concerns for children and parents, especially in dealing with rapid socio-cultural changes. Parental and adolescent empowerment can help them properly deal with challenges. The empowerment program should be based on increasing awareness, solutions for mutual understanding of expectations and beliefs of parents and adolescents from each other, and accountable interactions.

## Conclusion

 The finding of this study identified that families were dealing with problems in providing and improving the adolescents social health.

 The findings of this study identified that families were dealing with problems in providing and improving the social health of adolescents. Our findings also revealed key facilitators that empower the family to meet the social health needs of their adolescents. Politicians and health experts can use these results for planning and implementing interventions with the goal of family empowerment. There is a need for further studies on family empowerment approaches as a health-promoting setting for family members, in particular adolescents. It is also suggested to continue the qualitative studies on the social and cultural spaces affecting adolescent health, including schools, the media, and the cyberspaces.
